# A rice LSD1-like-type ZFP gene *OsLOL5* enhances saline-alkaline tolerance in transgenic *Arabidopsis thaliana*, yeast and rice

**DOI:** 10.1186/s12864-016-2460-5

**Published:** 2016-02-27

**Authors:** QJ Guan, HY Ma, ZJ Wang, ZY Wang, QY Bu, SK Liu

**Affiliations:** Key Laboratory of Saline-alkali Vegetation Ecology Restoration in Oil Field (SAVER), Ministry of Education, Alkali Soil Natural Environmental Science Center (ASNESC), Northeast Forestry University, No.26 Hexing Road, Nangang District, Harbin City, Heilongjiang 150040 China; Lab of Soybean Molecular Biology and Molecular Breeding, Northeast Institute of Geography and Agroecology, Chinese Academy of Sciences, No.138 Haping Road, Nangang District, Harbin City, Heilongjiang 150081 China

**Keywords:** Rice, LSD1-like gene family, Transcription factor, *OsLOL5*, *Arabidopsis thaliana*, Saline-alkaline, Yeast

## Abstract

**Background:**

Zinc finger proteins (ZFPs) play an important role in regulating plant responses to abiotic stress. However, little is known about the function of LSD1-like-type ZFP in saline-alkaline (SA) stress resistance of rice. In this study, *OsLOL5* (GenBank No. AJ620677), containing two LSD1-like-type C2C2 domains, was isolated and analyzed its protection roles in transgenic plants and yeast. *OsLOL5* was located in the nucleus as evidenced by the bombardment of onion epidermal cells.

**Results:**

*OsLOL5* expression significantly increased in rice leaves and roots under 150 mmol L-1 NaCl, 30 mM NaHCO_3_, and 10 mmol L-1 H_2_O_2_ treatment, respectively. Overexpression of *OsLOL5* in yeast resulted in SA tolerance at significant level. Transgenic Arabidopsis plants overexpressing *OsLOL5* grew well in the presence ofboth NaCl and NaHCO_3_ treatments, whereas wild-type plants exhibited chlorosis, stunted growth phenotype, and even death. SA stress caused significant changes in the malondialdehyde (MDA) contents in non-transgenic plants compared with those in transgenic lines. Transgenic rice overexpressing *OsLOL5* exhibited stronger resistance than NT under NaHCO_3_ treatment, as demonstrated by its greater shoot length, and fresh weight. The genes associated with oxidative stress, such as *OsAPX2*, *OsCAT*, *OsCu/Zn-SOD*, and *OsRGRC2*, were significantly upregulated in *OsLOL5*-overexpressing rice. The results suggested that *OsLOL5* improved SA tolerance in plants, and regulated oxidative and salinity stress retardation via the active oxygen detoxification pathway.

**Conclusions:**

The yeast INVScI bacterium grew significantly better than the control strain under NaCl, NaHCO_3_, and H_2_O_2_ treatments. These findings illustrated that *OsLOL5* overexpression enhanced yeast resistance for SA stress through active oxygen species. The present study showed that the *OsLOL5* genes involved in the ROS signaling pathways may combine with the model plant Arabidopsis and rice in LDS1-type ZFP by ROS signaling pathways that regulate cell necrosis. We speculated that the *OsLOL5* active oxygen scavenging system may have coordinating roles. The present study further revealed that *OsLOL5* ZFP could regulate oxidative stress function, but could also provide a basis for salt-resistant rice strains.

**Electronic supplementary material:**

The online version of this article (doi:10.1186/s12864-016-2460-5) contains supplementary material, which is available to authorized users.

## Background

Zinc finger proteins (ZFPs) are an important class of transcription factors. Zinc finger domains are the important feature of ZFPs, which consist of various numbers of cysteine (C) and histidine residues (H) combined with zinc ions [1]. Based on the number and location of these residues, ZFPs are classified into C2H2, C2HC, C2HC5, C3HC4, CCCH, C2C2, C4HC3, C6, and C8 groups [2]. Versatile ZFPs can bind DNA, RNA, proteins, and lipids to participate in the activities of an organism. C2H2 ZFPs are involved in different stages of plant growth and development and in various stress responses [3–9]. Plants possess a class containing the LSD1 zinc finger domain of the gene called the LSD1-like gene family, which is typically characterized by the presence of one to three LSD1-like zinc finger domains (C-X_2_-C-X_14_-C-X_2_-C). The LSD1-ke genes were found to be important in programmed cell death (PCD) and responses against diseases [1]. Furthermore, the *Arabidopsis* LSD1 gene responds to superoxide dismutase (SOD) signals, and suppresses PCD via AtLSD1 by upregulating the *Cu/Zn-SOD* gene to mit cell death proferation [10]. ZAT11, a zinc finger of *A. thaana*, is a dual-function transcriptional regulator that positively regulates primary root growth, but negatively regulates Ni21 tolerance [11]. The *TaLOL2* gene contains three typical LSD1-ke zinc finger domains. qRT—PCR analysis showed that *TaLOL2* is upregulated in early stripe rust infection, indicating the involvement of wheat stripe rust-induced defense responses [12].

To date, the ZFP transcription factor family in rice (*Oryza sativa* L.) has been a huge focus of stress research. After searching through the NCBI, Gramene, and Plant TFDB databases, we acquired the sequence data of 878 rice *ZFP* genes in 12 rice chromosomes. Among them, Chromosomes 1 and 11 contain 121 and 45 ZFP genes, respectively. Several ZTP gene functions have been characterized. A previous study showed that the *ZFP* gene results in certain yields and traits under abiotic stress related to cultivated rice [13]. For example, rice *ZFP245* is low-temperature and drought stress-related [14]. Overexpression of *ZFP182* in tobacco or rice increases tolerance for NaCl stress, thereby suggesting that C2H2-ZFP182 may be involved in the response of plants to salt stress [15]. Overexpression of *OsTOP6A1* increases tolerance for NaCl and mannitol stress in *A. thaliana* [16]. *OsZFP177* expression is induced during the cold season, drought, and upon exposure to H_2_O_2_ stress [17]. *OsLSD1* gene over-expression can accelerate the differentiation of the callus and promote chlorophyll b accumulation in transgenic plants. Antisense transgenic plants OsLSD1 are characterized by spontaneous necrotic lesions, enhanced disease resistance, and upregulated PR1 gene expression [1]. *OsLOL2* has two LSD1-ke zinc finger domains, and is important in rice growth and disease resistance. Over-expression of *OsLOL2* in transgenic rice significantly improves bacterial bight resistance. Over-expressing transgenic tobacco enhances bacterial wilt disease and *Pseudomonas syringae* resistance [18, 19]. However, studies on salt tolerance in the gene *OsLOL5* remain mited to date. In the present study, the *OsLOL5* gene from rice leaves of cultivar LongJing11 (LJ11) was cloned using RT-PCR, and the mRNA expression levels under SA treatment were detected by using qRT-PCR and transgenic techniques. This study aimed to reveal the mechanisms of the *OsLOL5* gene in regulating rice responses to SA stress.

## Methods

### Plant materials and stress treatment

*A. thaliana* (ecotype: Columbia) seeds for SA treatment were provided by the Environmental Research Center of Northeast Forestry University, Harbin City, Heilongjiang Province, China. *a*ast Institute of Geography and Agroecology, Chinese Academy of Sciences, Harbin City, Heilongjiang province, China. Nine-day-old rice seedlings were used for 150 mmol L^-1^ NaCl, 30 mmol L^-1^ NaHCO_3_, and 5 mmol L^-1^ H_2_O_2_ stress treatments. Leaf and root samples were collected after treatment and immediately frozen in quid nitrogen. RNA was extracted using an RNeasy Plant Mini Kit (Qiagen, Dusseldorf, Germany) and then stored at –80 °C in the Northeast Institute of Geography and Agroecology, Chinese Academy of Sciences, Harbin City, Heilongjiang Province, China. Each stress treatment was repeated six times.

### Cloning of OsLOL5 gene

The full-length *OsLOL5* cDNA sequence was obtained by RT-PCR using primer pair OsLOL5-P1, which was designed based on the gene sequence in GenBank (AJ620677, http://www.ncbi.nlm.nih.gov/nuccore/40809630?report=genbank). The total RNA was isolated from four leaves from rice seedlings of LJ11 using Trizol (Invitrogen, Carlsbad, CA, USA) according to the manufacturer’s instructions. First-strand cDNA was synthesized using a *SuperScript*^III^ reverse transcriptase kit. The specific primer pair OsLOL5-P1 was designed with Primer Premier 5.0 (Premier Biosoft, Palo Alto, USA) and used for full-length ampfication of the gene, which was cloned into the pMD18-T(Takara Biotechnology in DAAN) vector and confirmed through sequencing (Invitrogen, Shanghai, China).

### Subcellular localization of OsLOL5 by transient expression in onion epidermal tissue

**Fig. 1:**
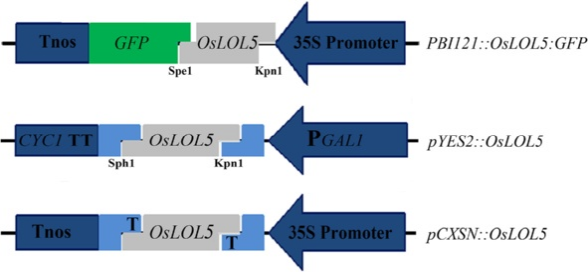
Schematic of the expression vectors *PBI121::OsLOL5::GFP*, *pCXSN::OsLOL5*, and *pYES2::OsLOL5*. 35S Promoter, Cauliflower Mosaic Virus 35S Promoter; Tnos, nos-terminator; P*GAL1*, Galactokinase promoter; *CYC1* TT, CYC1 terminator; and GFP, green fluorescent protein

To determine the subcellular location of the OsLOL5 protein, the PCR product generated by primer pair OsLOL5-P2 was used to construct the *PBI121::OsLOL5::GFP* expression vector (Fig. 1). The *PBI121-OsLOL5::GFP* fusion plasmids were coated onto 20 ml of 50 mg · mL^−1^ gold particles with 2.5 M CaCl_2_ and 0.1 mol L^-1^ spermidine and mixed rigorously using a vortex for 2 min. Plasmid-coated particles were dehydrated using 75 and 95 % ethanol prior to bombardment. Single-layer epidermal sheaths peeled from onion bulbs were placed on 1/2 MS plates and subjected to particle bombardment using the standard procedure provided by the manufacturer. Plasmid-coated gold particles were accelerated with a helium burst at 1100 psi in a PDS-1000/He instrument (Bio-Rad, Hercules, California, USA). Plates containing transfected onion tissues were wrapped in foil and incubated in the dark overnight (16–20 h) at room temperature [20].

### OsLOL5 gene expression analysis

Total RNA was extracted from roots and leaves after treatment of the seedlings with 150 mmol L^-1^ NaCl, 30 mmol L^-1^ NaHCO_3_, or 5 mmol L^-1^ H_2_O_2_ after different periods of time (0 h as control group, 12, 24, and 48 h), respectively. First-strand cDNA was synthesized from 1 μg of total RNA with SuperScript®III reverse transcriptase and oligo-dT primers (Invitrogen, Shanghai, China) according to the manufacturer’s instructions. cDNA was diluted with nuclease-free water to 1:10. Aliquots of the same cDNA sample were used for real-time PCR with primer pair OsLOL5*-*P3. The Os*Actin1* gene was used as an internal control. PCR was performed in a 25 μL reaction mixture containing 400 nmol L^-1^ of each primer, 1 × SYBR Green qPCR master mix (Agilent Technologies, Santa Clara, CA, USA), and approximately 30 ng of cDNA. Real-time PCR was performed on the Agilent Stratagene MxPro-Mx3000p (Agilent Technologies, Santa Clara, CA, USA) using the following procedure: 30 s at 95 °C for denaturation, followed by 40 cycles of 5 s at 94 °C, 30 s at 60 °C, and 40s at 72 °C. Relative transcript abundance was calculated according to the manufacturer’s instructions. The specificity of each primer pair was verified by determining the melting curves at the end of each run, and sequencing the amplified bands from gel electrophoresis.

### Analysis of sensitivity to abiotic stress in OsLOL5 transgenic yeast

To detect *OsLOL5* response to NaCl, NaHCO_3_, and H_2_O_2_, *OsLOL5* was amplified using primer pair OsLOL5-P4, and the PCR product was cloned into pMD18-T. OsLOL5 was cut from pMD18-T with restriction endonucleases KpnI and SphI, and ligated into the yeast expression vector pYES2. Using the LiAc method, the plasimds *pYES2* and *pYES2::OsLOL5* (Fig. 1) were transformed into the *Saccharomyces cerevisiae* strain INVSc1.Transformed yeast strains were grown in synthetic defined medium minus the appropriate amino acids (SD-Ura) for selective growth for the expression plasmids. To analyze abiotic stress tolerances, the *pYES2-* and *pYES2::OsLOL5-*transformed cell cultures were adjusted to an OD_600_ of 0.6 using yeast extract-peptone-dextrose (YPD) medium. Ten-fold serial dilutions of yeast strains were prepared, and 5-μl aliquots of each dilution were spotted on solid YPD medium containing NaCl (0 mol L^-1^ as control group, 0.8, and 1 mol L^-1^), NaHCO_3_ (30, 32, and 40 mmol L^-1^), or H_2_O_2_ (3, 3.2, and 3.4 mmol L^-1^). All of the plates were incubated at 30 °C for 3–6 days.

### Functional analysis of OsLOL5 in Arabidopsis

Using primer pair OsLOL5-P4, the *OsLOL5* PCR product was ligated into the expression vector pCXSN after XcmI digestion [21]. For *Arabidopsis* transformation, the *pCXSN::OsLOL5* (Fig. 1) vectors were first introduced into *Agrobacterium tumefaciens* GV3101 by electroporation. *Arabidopsis* cv Col-0 plants were transformed via floral dip method as previously described [22]. *Arabidopsis* transgenic seeds were plated on half-strength Murashige and Skoog (MS) medium containing 25 mg · L^−1^ hygromycin for selection. Resistant plants were used for molecular identification. To study the function of OsLOL5 in the abiotic stress response, the transgenic T3 generation encoding *OsLOL5* driven by the cauliflower mosaic virus (CaMV35S) promoter were tested with primer pair OsLOL5-P5 and then used for the following studies. The T3 (#1–#3) and WT seeds were sterilized and sowed in 1/2 MS medium for germination for 10 d. The seedlings were then transferred to 1/2 MS medium containing 0 (0 mmol L^-1^ as control group), 100, 125, or 150 mmol L^-1^ NaCl or 0, 2, 4, and 6 mmol L^-1^ NaHCO_3_. After 30 d, the growth phenotype, plant height, fresh weight, and MDA content of the seedlings were measured.

### Alkaline stress tolerance analysis of OsLOL5 in rice

For rice transformation, the pCXSN*::*OsLOL5 vectors were transferred into *A. tumefaciens* EHA105 through electroporation. *OsLOL5* was transformed into *O. sativa* L. ssp. *japonica* cv. “Longjing 11” by using the *Agrobacterium-*mediated co-cultivation method. The transgenic T2 generation encoding OsLOL5 driven by the cauliflower mosaic virus (CaMV35S) promoter were tested with primer pair OsLOL5-P5. OsOLO5 expression in transgenic plants was confirmed by using Northern blot. Three independent T2 homologous transgenic lines and the control Longjing 11 were used for alkaline stress tolerance experiments. For alkaline treatment, concurrent buds were transferred to the stress liquid culture medium containing 0 (0 mmol L^-1^ as control group), 5, 7.5, and 10 mmol L^-1^ NaHCO_3_. After 21 d, the growth phenotype, root length, fresh weight, and MDA content of seedlings were measured. Simultaneously, the expression level of oxidative stress response genes *OsAPX2* (AB053297), *OsCAT* (AB020502), *OsCu/Zn-SOD* (AK059841), and *OsRGRC2* (AY136765) were analyzed. Procedures for RNA extraction and real-time PCR were similar to those described and listed in Table 1.

**Table 1:** The primers used in gene clone and qRT-PCR

Primer name	Forward (5’-3’)	Reverse (5’-3’)
OsLOL5-P1	GATGTCTCAGCTACCACTTGCA	GGTCACCTTTCCTGTCTACAT
OsLOL5-P2	GGTACCATGTCTCAGCTACCACTTGC	ACTAGTGGCTTCAGCTAGCCCTGAT
OsLOL5-P3	GCAACCCACAAGAACTAACTCATC	GGCTTGTCCATACCATCTTGAAC
OsActin1	CTTCATAGGAATGGAAGCTGCGGGTA	CGACCACCTTGATCTTCATGCTGCTA
OsLOL5-P4	GGTACCATGTCTCAGCTACCACTTGC	GCAACCCACAAGAACTAACTCATC
OsLOL5-P5	GGTACCATGTCTCAGCTACCACTTGC	ATCGGGGAAATTCGCTAGTG
OsAPX2P1	TCCTACGCCGACTTCTACCA	CGGCGTAATCCGCAAAGAAG
OsCATP1	TACTTCCCATCCCGCTACGA	TCCTTACATGCTCGGCTTCG
OsCu/Zn-SODP1	CAGGTTGAGGGAGTCGTCAC	GGTTGCCTCAG CTACACCTT
OsRGRC2P1	GGCCAGCCAACTAAACCTGA	CCAGCATAACAACCGCACAC

### MDA content measurements

MDA content was determined using the previously described thiobarbituric acid reaction [23]. Absorbance levels at 532 and 600 nm were determined using a spectrophotometer. After subtracting the non-specific absorbance at 600 nm, MDA concentration was determined using its extinction coefficient 155 mM^−1^ · cm^−1^.

### Data analysis

Analysis of variance (AVONA) and multiple comparison by software data processing system (DPS) (version 7.05).

## Results

### OsLOL5 is a LSD1-like zinc finger gene

*OsLOL5* was successfully cloned using RT-PCR with specific primer pair OsLOL5-P1 from *O. sativa* L. ssp. cv. “LongJing 11” with alkaline stresses then inserted into the pMD18-T vector. Sequencing results confirmed that the OsLOL5 sequence was identical to GenBank No. AJ620677. Sequence analysis showed that the full-length sequence encoded 163 amino acids, with predicted molecular mass and isoelectric point of 17.6 kDa and 6.03, respectively. Based on structural properties indicated by SMART programs, the predicted protein contains two LSD1-like zinc finger domains. The LSD1-like zinc finger domains contain the sequence C-X_2_-C-X_14_-C-X_2_-C, where X can be any amino acid. The subscripts indicate the number of residues. Homology alignment analysis using ClustalX software showed that the deduced amino acid sequences were highly similar to several previously isolated LSD1-like ZFPs in *Arabidopsis* and rice (Additional file 1: Figure S1). We also found that *OsLOL5* was clustered in the same group with *AtLOL2* because they share the highest similarity in terms of identities. Therefore, OsLOL5 was determined to be an LSD1-like ZFP.

### OsLOL5 localized in the nucleus

**Fig. 2:**
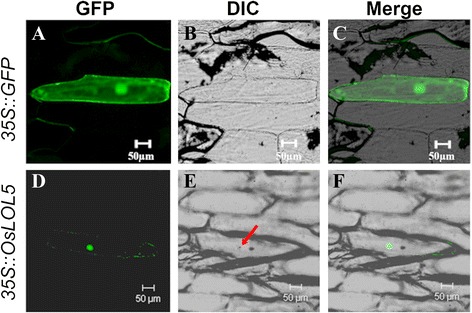
Subcellular localization of the 35S::OsLOL5::GFP fusion protein. **a**, **d** GFP, green fluorescence. **b**, **e** DIC, bright field. **c**, **f** Merge, green fluorescence, and bright field superposition. Nucleus is marked with the red arrow

Online analysis tool Psort predicted that OsLOL5 would localize in the nucleus and cytoplasm with 47.8 and 34.8 % probability, respectively. To verify the prediction, a *pBI121::OsLOL5::GFP* plasmid was transformed into onion epidermal cells using gene gun bombardment. Cells were cultured for 18–24 h in the dark, and the transformed onion epidermal cells were observed for GFP signals using laser confocal fluorescence microscopy. When driven by promoter 35S, fusion protein *OsLOL5::GFP* was expressed in onion epidermal cells with green fluorescence in the nucleus, thereby suggesting that OsLOL5 localized in the nucleus (Fig. 2).

### Expression pattern of OsLOL5 under abiotic stress treatments

**Fig. 3:**
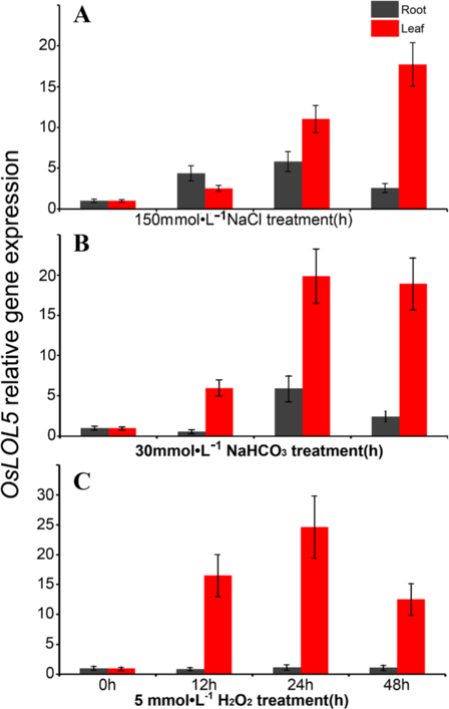
Real-time quantitative expression analysis of the *OsLOL5* gene under **a** salt (150 mmol L^-1^ NaCl), **b** alkali (30 mmol L^-1^ NaHCO_3_), and **c** oxidative (5 mmol L^-1^ H_2_O_2_) stress treatments, respectively

To obtain an overview of the *OsLOL5* expression pattern under different abiotic stress conditions, qRT-PCR was performed to examine its transcript in rice after saline, alkaline, and oxidative stress treatments. *OsLOL5* expression was significantly induced in both leaves and roots by 150 mmol L^-1^ NaCl treatment. The maximum 17.7-fold increase compared with the untreated control in leaves occurred 48 h after treatment, but a 5.8-fold increase compared with the control in roots occurred 24 h after treatment (Fig. 3a). *OsLOL5* was upregulated in leaves and roots subjected to 30 mmol L^-1^ NaHCO_3_ treatment and peaked after 24 h. *OsLOL5* expressions were 19- and 6-fold higher than the untreated control in leaves and roots, respectively (Fig. 3b). *OsLOL5* expression dramatically increased by 25-fold in leaves after 24 h of 5 mmol L^-1^ H_2_O_2_ treatment compared with the untreated control (Fig. 3c). However, H_2_O_2_ treatment had no significant effect in *OsLOL5* expression in roots. *OsLOL5* was a stress-responsive ZFP, and exhibited differential expression patterns in leaves and roots under SA treatments.

### OsLOL5 improved yeast tolerance to NaHCO_3_ and H_2_O_2_

**Fig. 4:**
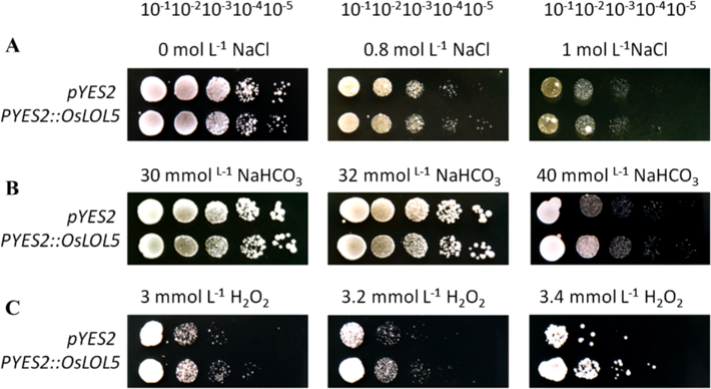
Analysis of the sensitivity of yeast expressing pYES2::OsLOL5 to **a** (0, 0.8, and 1.0 mol · L^−1^ NaCl); **b** (30, 32, and 40 mmol · L^−1^ NaHCO_3_); and **c** (3, 3.2, and 3.4 mmol · L^−1^ H_2_O_2_). The mean dilution rates of YPD were 10^0^, 10^−1^, 10^−2^, 10^−3^, 10^−4^, and 10^−5^

To gain a preliminarily understanding of the function of *OsLOL5* in abiotic stress, we surveyed growth characteristics of INVSc1 yeast containing pYES2 or pYES2-OsLOL5 under the following stresses: 0, 0.8, and 1 mol · L^−1^ NaCl; 30, 32, and 40 mmol · L^−1^ NaHCO_3_; and 3, 3.2, and 3.4 mmol · L^−1^ H_2_O_2_ stress. INVSc1 yeast cells containing pYES2 or pYES2-OsLOL5 were cultured in YPD + galactose media plates supplied with different concentrations of NaCl, NaHCO_3_, and H_2_O_2_. Growth conditions were observed after 72 h of incubation at 30 °C (Fig. 4). As NaHCO_3_ and H_2_O_2_ concentrations increased, yeast transformed with pYES2-OsLOL5 exhibited better growth conditions than control yeast transformed with pYES2. More clones were clearly present in the pYES2-OsLOL5-transformed yeast, particularly in 40 mmol · L^−1^ NaHCO_3_ and 3.4 mmol · L^−1^ H_2_O_2_, than in the control pYES2-transformed yeast after dilutions of 10^−2^ and 10^−3^. However, growth conditions between YES2 and YES2-OsLOL5 were not significantly different under NaCl treatment. Thus, yeast cells expressing *OsLOL5* were more resistant to NaHCO_3_ and H_2_O_2_ stress.

### OsLOL5 overexpression in A. thaliana enhanced SA stress tolerance

To investigate the biological function of *OsLOL5* in plants, we overexpressed *OsLOL5* in *Arabidopsis* under the control of promoter CaMV 35S. PCR screening results showed that the OsLOL5 band was detected in five T1 (#1–#5) lines (Additional file 2: Figure S2A), whereas the negative control did not amplify the target gene fragment. These results indicated that the *OsLOL5* gene was inserted into the *Arabidopsis* genome. Furthermore, Northern blot analysis of the T3 transgenic lines derived from the five identified T1 lines confirmed that OsLOL5 was successfully overexpressed in the *Arabidopsis* genome (Additional file 2: Figure S2B). Three T3 lines, namely, T3-#1, -#2, and -#3 seedlings, were used for further analysis.

**Fig. 5:**
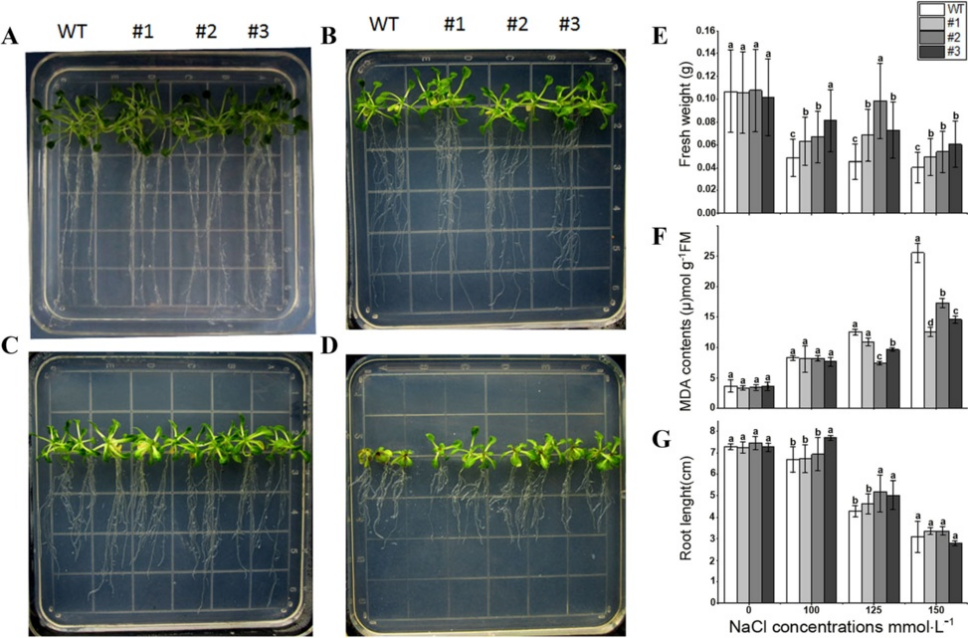
Analysis of *OsLOL5* transgenic plants to NaCl treatment. **a**, **b**, **c**, and **d** show phenotypes of *OsLOL5* transgenic and WT lines subjected to 0, 100, 125, or 150 mM NaCl treatment, respectively. **e**, **f**, and **g** show fresh weight, endogenous MDA levels, and root length changes in *OsLOL5* transgenic and WT lines, respectively

To identify the function of OsLOL5 in stress response, the T3 generation *OsLOL5*-overexpressing lines were subjected to SA tolerance assay. Under standard culture conditions, no noticeable difference was observed between transgenic lines overexpressing OsLOL5 and non-transformed plants. After 14 d of NaCl treatment, both WT and transgenic plants experienced growth retardation in a dose-dependent manner. However, retardation was more apparent in WT plants than in transgenic plants (Fig. 5a, b,c, and d). Moreover, a significant and dramatic difference in fresh weight and root length between the transgenic and WT plants was noted (Fig. 5e, g). Salt stress can cause oxidative damage to cell membranes. MDA content is an indicator of oxidative stress. In the present study, MDA levels in transgenic lines decreased significantly compared with those in WT plants (Fig. 5f).

**Fig. 6:**
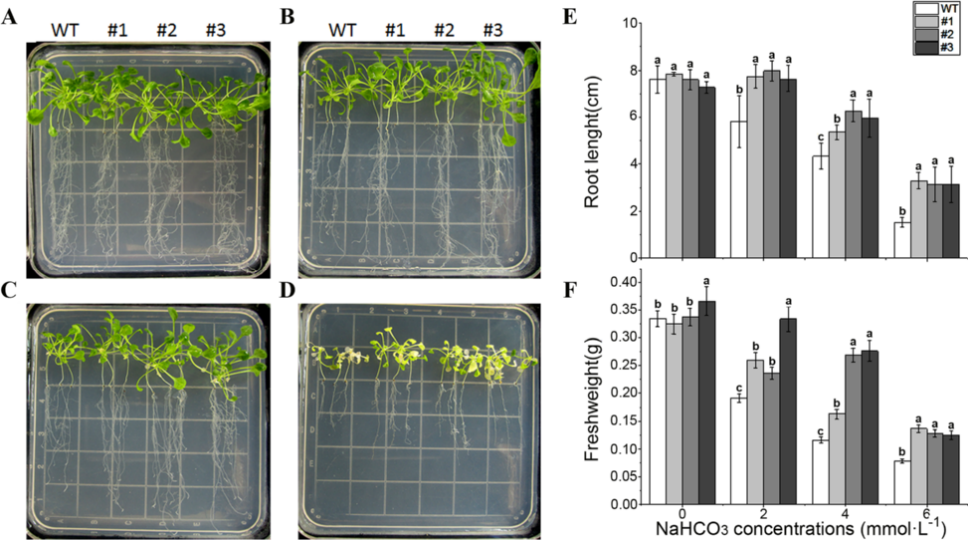
Analysis of *OsLOL5* transgenic plants under NaHCO_3_ treatment. **a**, **b**, **c**, and **d** show phenotypes of *OsLOL5* transgenic and WT lines subjected to 0, 2, 4, and 6 mmol · L^−1^ NaHCO_3_ treatment, respectively. **e** and **f** show root length and fresh weight in *OsLOL5* transgenic and WT lines, respectively

Similarly, after 14 d of treatment, NaHCO_3_ significantly inhibited both transgenic and WT lines (Fig. 6), but the fresh weight of the transgenic lines was significantly higher than that of the WT controls. At high NaHCO_3_ concentrations (4 mmol · L^−1^ and 6 mmol · L^−1^), both WT and transgenic plants exhibited chlorosis, but the fresh weight and root length in WT was significantly lower than in *OsLOL5* transgenic lines. These results indicated that *OsLOL5* played an important role in the stress response, and increased the SA stress tolerance of plants.

### OsLOL5 overexpression in rice increased alkaline stress tolerance

**Fig. 7:**
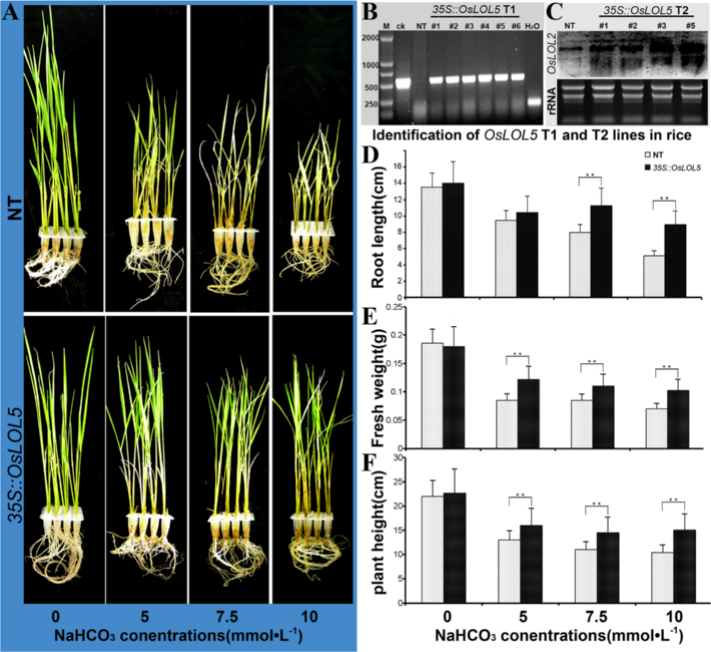
Analysis of *OsLOL5* transgenic rice under NaHCO_3_ treatment. **a** shows phenotypes of the *OsLOL5* transgenic and NT lines subjected to 0, 5, 7.5, or 10 mmol · L^−1^ NaHCO_3_ treatment. **b** and **c** show identification of *OsLOL5* T1 and T2 lines in rice. **d**, **e**, and **f** show root length, fresh weight, and plant height in *OsLOL5* transgenic and NT lines. ** means significant level (*p* < 0.01) by T-test

The *OsLOL5* gene was introduced in *O. sativa* L. ssp. cv. “Longjing 11” via *Agrobacterium-*mediated transformation under the control of promoter CaMV 35S. Six T1 generation (#1–#6) *OsLOL5* transgenic lines were identified by PCR (Fig. 7b). To detect *OsLOL5* expression in transgenic rice lines, Northern blot was performed on young leaves of transgenic and NT rice. *OsLOL5* overexpression in different levels was observed in T2 transgenic rice lines #1, #2, #3, and #5 (Fig. 7c). Responses of transgenic rice lines (#1–#3) and NT plants to NaHCO_3_ stress were determined to further investigate the roles of *OsLOL5* in rice. Under standard culture conditions, no noticeable difference between transgenic lines and NT plants was observed. After 21 d of NaHCO_3_ treatment, NT plants exhibited growth retardation and chlorosis, whereas *OsLOL5* overexpression lines exhibited continuous growth and remained green (Fig. 7a). Moreover, a marked difference in both root length, plant height and fresh weight was observed between transgenic and WT plants (Fig. 7d, e, f). These results further indicated that *OsLOL5* played an important role in stress response and increased the NaHCO_3_ stress tolerance of plants.

### Expression of oxidative stress response genes were enhanced in OsLOL5-overexpressing rice

**Fig. 8:**
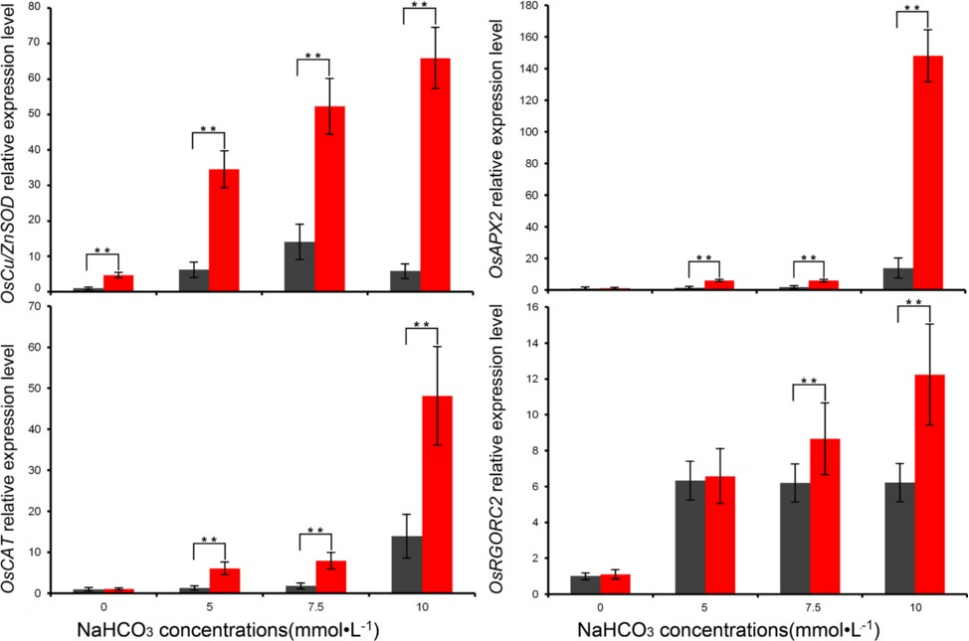
Expression of stress-related genes in *OsLOL5-*overexpressing and NT rice. Total RNA was extracted from rice seedlings at the four-leaf stage grown under control and salt treatments. The transcript levels of *OsAPX2, OsCAT, OsCu/Zn-SOD,* and *OsRGRC2* were measured by quantitative real-time PCR under unstressed conditions or 0, 5, 7.5, and 10 mmol · L^−1^ NaHCO_3_ for 21 d, respectively. Data are written as means and standard errors of three replicates. ** show significant differences at *p* < 0.01 (means ± SD, *n* =6)

SA stress can cause oxidative stress, and OsLOL5-overexpressing lines show significant alkaline stress tolerance. To verify whether the expression of oxidative stress response genes is also enhanced in these transgenic lines, several oxidative stress response genes (*OsAPX2*, *OsCAT*, *OsCu/Zn-SOD*, and *OsRGRC2*) were chosen and compared between NT and *OsLOL5*-overexpressing lines in response to NaHCO_3_ treatment. Real-time PCR assay indicated that NaHCO_3_ stress induced the expression of these genes, and the expression level of oxidative stress response genes in *OsLOL5*-overexpressing lines was significantly higher than in NT plants (Fig. 8). The expression of OsCu/Zn-SOD genes also were upregulated in the absence of salt stresses. Without NaHCO_3_ treatment, the expressions of most oxidative stress response genes (*OsAPX2*, *OsCAT*, and *OsCu/Zn-SOD*) were still notably higher in the *OsLOL5*-overexpressing lines than in NT plants. This finding indicated that *OsLOL5* overexpression promoted the constitutive expression of oxidative stress response genes. Therefore, higher expression of oxidative stress response genes might also contribute to enhanced stress tolerance of *OsLOL5*-overexpressing lines.

## Discussion

Plant growth and development may be influenced by biotic and abiotic stress, such as diseases, insects, low temperatures, drought, high salinity, and wounding. Plants have developed many complicated signal transduction and regulatory mechanisms to adapt to environmental changes and to continue to grow and develop under such harsh conditions [24]. ZFP expression can be induced by the cold season, drought, and H_2_O_2_ stress. The OSISAP1ABA gene from Indian rice coding C2H2 ZFP is expressed under stress conditions, such as high salt, low temperature, and drought; this behavior indicates the relationship between *OsISAP1* and abiotic stress [25]. In the present study, the *OsLOL5* gene was cloned from rice cv. “Longjing11”, which contains two LSD1-like zinc finger domains and shares a high homology with AtLOL2 (Additional file 1: Figure S1). This gene has a typical C-X2-C-X14-C-X2-C structure (Additional file 1: Figure S1). OsLOL2 not only participated in growth development, but was also affected by pathogenic microorganism stress. *OsLOL2* overexpression in tobacco enhances resistance to bacterial wilt and *P. syringae* [18]. *OsLOL5* gene expression increased in “Longjing11” leaf and Longjing root with SA stress (Fig. 3), thereby indicating that *OsLOL5* was a broad spectrum-resistant transcription factor. Stress is usually accompanied with high salinity and numerous reactive oxygen species, which can lead to lipid peroxidation of the cell membrane in plants, mutation, DNA strand breaks, and protein damage. *OsLOL5* gene expression increased with H_2_O_2_ stress in rice leaf, which revealed that OsLOL5 may respond to oxidation stress. Future studies may consider predicting which gene can regulate expression to improve tolerance for salinity stress. Experiments confirmed that “Longjing11” *OsLOL5* localized and functioned in the nucleus (Fig. 3). Yeast grows quickly, and its experiment cycle is shorter than in plants; when galactosum is induced, it exhibits high expression efficiency [26]. The present study examined the expression of the GAL1 promoter [27, 28] to establish the yeast INVScI OsLOL5, and enhanced resistance to SA stress (Fig. 4). The yeast INVScI bacterium grew significantly better than the control strain under NaCl, NaHCO_3_, and H_2_O_2_ treatments, particularly under 3.2 mM H_2_O_2_ stress. These findings illustrated that *OsLOL5* overexpression enhanced yeast resistance for SA stress through active oxygen species.

MPKs can directly modulate *ZAT10* gene expression through the phosphorylation of transcription factors [29]. Thirty-four (34) *Medicago* CCCH Zinc finger genes have been identified in response to PEG-6000, NaCl, and ABA stress conditions [30]. The LSD1-like family is a multi-resistance gene family, although studies have shown that transcription factor OsLOL2 is involved in rice growth and disease resistance [31]. *Arabidopsis*-transferred *OsLOL5* strains showed resistance after 14 d compared with WT grown at 100, 125, and 150 mmol · L^−1^ NaCl stress. Under stress treatment, the MDA content of transgenic lines was lower than that of WT. OsLOL5 may be involved in photosynthesis because overexpression strains have high chlorophyll contents. Overexpression of rice also showed resistance to alkaline salts of NaHCO_3_ (Fig. 8). Overexpression of Longjing11 rice line under 7.5 mmol · L^−1^ and 10 mmol · L^−1^ stress resulted in significantly higher (*p* < 0.01) height, fresh weight, and chlorophyll content than Longjing 11. QRT-PCR detection of rice treated with NaHCO_3_ showed that *OsAPX2, OsCAT, OsCu/Zn-SOD,* and *OsRGRC2* genes were transcribed. These genes were induced in both transgenic and NT lines, but the increased rate in transgenic lines was much higher than in NT. The highest expression level was observed in OsAPX2, which was approximately 100-fold of the expression in untreated NT. These results were consistent with results on *Arabidopsis* AtLSD1 and AtLOL1, which were controlled by negative and positive ROS-mediated signaling pathways, respectively [32]. The present study showed that the *OsLOL5* genes involved in the ROS signaling pathways may combine with the model plant *Arabidopsis* and rice in LDS1-type ZFP by ROS signaling pathways that regulate cell necrosis [12]. We speculated that the OsLOL5 active oxygen scavenging system may have coordinating roles. The present study further revealed that OsLOL5 ZFP could regulate oxidative stress function, but could also provide a basis for salt-resistant rice strains.

## Conclusion

The yeast INVScI bacterium grew significantly better than the control strain under NaCl, NaHCO_3_, and H_2_O_2_ treatments. These findings illustrated that *OsLOL5* overexpression enhanced yeast resistance for SA stress through active oxygen species. The present study showed that the *OsLOL5* genes involved in the ROS signaling pathways may combine with the model plant Arabidopsis and rice in LDS1-type ZFP by ROS signaling pathways that regulate cell necrosis. We speculated that the *OsLOL5* active oxygen scavenging system may have coordinating roles. The present study further revealed that *OsLOL5* ZFP could regulate oxidative stress function, but could also provide a basis for salt-resistant rice strains.

## Additional files

Additional file 1: Figure S1. Homology alignment of OsLOL5 protein with other LOL proteins from Arabidopsis and Rice. Zf-LSD1:C4-zinc finger domain was marked with *. OsLOL2 (LOC_Os12g41700), OsLOL3 (Q6ASS2), AtLSD1 (At4g20380), OsLOL4 (Q84UR0), OsLSD1 (LOC_Os08g06280), AtLOL1 (At1g32540), OsLOL5 (AJ620677), AtLOL2 (At4g21610). (JPG 2330 kb) Additional file 2: Figure S2. PCR analysis of *OsLOL5*-overexpressing T1 and T3 strains. Northern hybridization of (A) WT: wild-type *Arabidopsis thaliana*; #1–#5: T1 *OsLOL5-*overexpressing *A. thaliana* strain; (B) WT: wild-type *A. thaliana*; #1–#3: T3 *OsLOL5*-overexpressing *A. thaliana* strain. (JPG 55 kb)

## Abbreviations

ABA: Abscisic acid
CaMV: Cauliflower mosaic virus
LJ11: LongJing11
MDA: Malondialdehyde
MS: Murashige and Skoog
NT: Non-transgenic rice
SA: Saline-alkaline
WT: Wild type
ZFPs: Zinc finger proteins
