# Supplementary figures and images for: A rice LSD1-like-type ZFP gene OsLOL5 enhances saline-alkaline tolerance in transgenic Arabidopsis thaliana, yeast and rice
Source: BMC Genomics. 2016 Feb 27;17:142. doi: 10.1186/s12864-016-2460-5 (PMC4769587; doi:10.1186/s12864-016-2460-5)

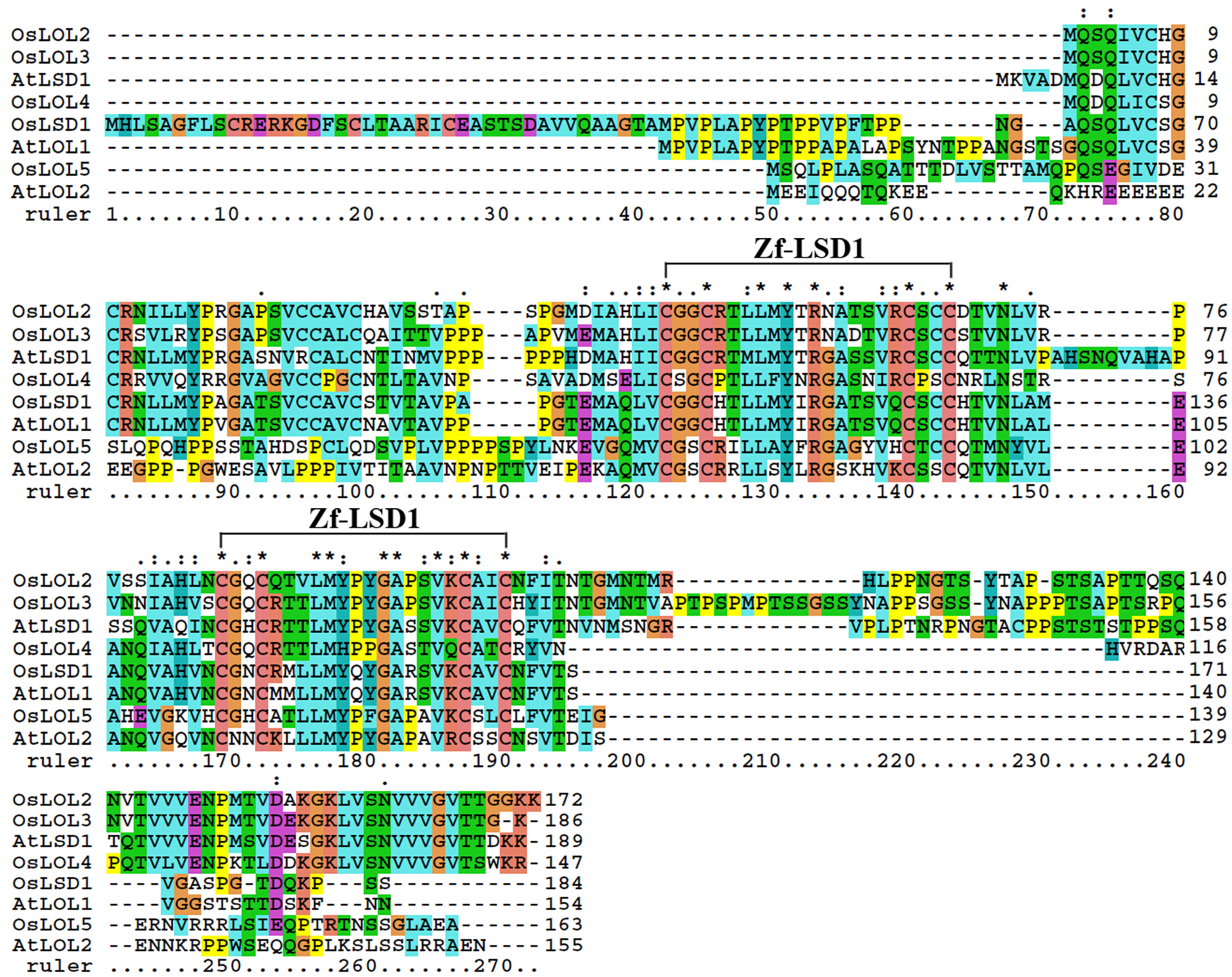

Supplement: Additional file 1: Figure S1. — Homology alignment of OsLOL5 protein with other LOL proteins from Arabidopsis and Rice. Zf-LSD1:C4-zinc finger domain was marked with *. OsLOL2 (LOC_Os12g41700), OsLOL3 (Q6ASS2), AtLSD1 (At4g20380), OsLOL4 (Q84UR0), OsLSD1 (LOC_Os08g06280), AtLOL1 (At1g32540), OsLOL5 (AJ620677), AtLOL2 (At4g21610). (JPG 2330 kb) [file 12864_2016_2460_MOESM1_ESM.jpg]

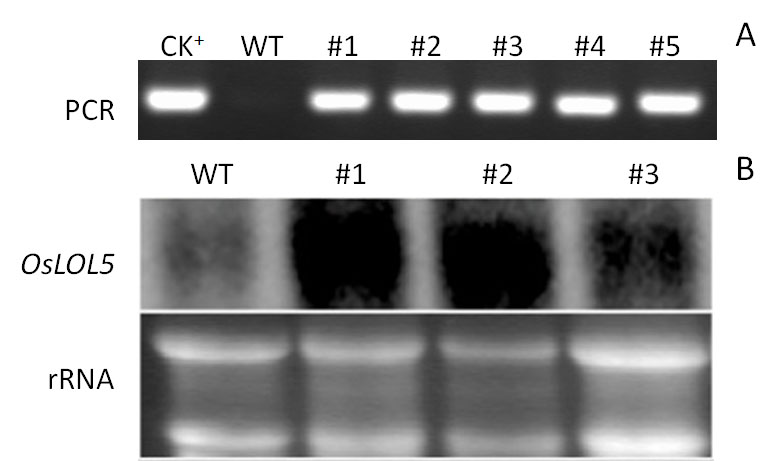

Supplement: Additional file 2: Figure S2. — PCR analysis of OsLOL5-overexpressing T1 and T3 strains. Northern hybridization of (A) WT: wild-type Arabidopsis thaliana; #1–#5: T1 OsLOL5-overexpressing A. thaliana strain; (B) WT: wild-type A. thaliana; #1–#3: T3 OsLOL5-overexpressing A. thaliana strain. (JPG 55 kb) [file 12864_2016_2460_MOESM2_ESM.jpg]
